# Characterization of Home Care Services and care for children with special health care needs^*^

**DOI:** 10.1590/1980-220X-REEUSP-2022-0032en

**Published:** 2022-07-18

**Authors:** Diana Augusta Tres, Rafael Gue Martini, Beatriz Rosana Gonçalves de Oliveira Toso, Elisangela Argenta Zanatta

**Affiliations:** 1 Universidade do Estado de Santa Catarina, Chapecó, SC, Brazil.; 2 Universidade do Estado de Santa Catarina, Florianópolis, SC, Brazil.; 3 Universidade Estadual do Oeste do Paraná, Cascavel, PR, Brazil.

**Keywords:** Comprehensive Health Care, Home Nursing, Home Care Services, Chronic Disease, Nursing, Atención Integral de Salud, Atención Domiciliaria de Salud, Servicios de Atención de Salud a Domicilio, Enfermedad Crónica, Enfermería, Assistência Integral à Saúde, Assistência Domiciliar, Serviços de Assistência Domiciliar, Doença Crônica, Enfermagem

## Abstract

**Objective:**

To characterize Home Care Services in the state of Santa Catarina, Brazil, and to know the care demands of children with special health care needs.

**Method:**

Descriptive, exploratory study with a quantitative approach. Data collection carried out through a questionnaire, via *Google Forms,* with professionals from seven Home Care Services, from April to July 2020. Data were organized in the software Excel and analyzed through descriptive statistics with frequency and percentage distribution.

**Results:**

Of the seven services participating in the study, in 85.7% the nurse is the teams’ coordinator. Opening hours in most services are during the day, on weekdays and weekends, and all of them receive referrals from Primary Health Care teams. Regarding care demands, all children need psychomotor and social rehabilitation, 72.2% use oxygen therapy, 66.6% gastrostomy, 55.5% tracheostomy, and 50% mechanical ventilation.

**Conclusion:**

Home care presents complexities due to chronic conditions and the use of technological devices. Thus, home care teams are fundamental in the assistance and training of family members/caregivers for children home care.

## RESUMO

**Objetivo:**

Caracterizar os Serviços de Atenção Domiciliar no estado de Santa Catarina e conhecer as demandas de cuidado das crianças com necessidades especiais de saúde.

**Método:**

Estudo descritivo, exploratório, de abordagem quantitativa. Coleta de dados realizada por meio de questionário, via *Google Forms*, com profissionais de sete Serviços de Atenção Domiciliar, de abril a julho de 2020. Os dados foram organizados no programa *Excel* e analisados pela estatística descritiva com distribuição de frequência e percentual.

**Resultados:**

Dos sete serviços que participaram do estudo, em 85,7% o enfermeiro é o coordenador das equipes. O horário de funcionamento na maioria dos serviços é diurno, em dias úteis e finais de semana e todos recebem encaminhamentos de equipes da Atenção Primária à Saúde. Em relação às demandas de cuidados, todas as crianças necessitam de reabilitação psicomotora e social, 72,2% fazem uso de oxigenoterapia, 66,6% de gastrostomia, 55,5% de traqueostomia e 50% de ventilação mecânica.

**Conclusão:**

O cuidado domiciliar apresenta complexidades em decorrência de condições crônicas e uso de dispositivos tecnológicos. Assim, equipes de atenção domiciliar são fundamentais na assistência e capacitação de familiares/cuidadores para o cuidado domiciliar às crianças.

## RESUMEN

**Objetivo:**

Caracterizar los Servicios de Atención Domiciliar en el estado de Santa Catarina y conocer las demandas de cuidado a los niños con necesidades especiales de salud.

**Método:**

Estudio descriptivo, exploratorio, de abordaje cuantitativo. Colecta de datos realizada por medio de cuestionario, vía *Google Forms*, con profesionales de siete Servicios de Atención Domiciliar, de abril a julio de 2020. Los datos fueron organizados en el Programa *Excel* y analizados a través de estadística descriptiva con distribución de frecuencia y porcentaje.

**Resultados:**

De los siete servicios que participaron del estudio, en el 85,7% de ellos el enfermero es el coordinador de los equipos. El horario de en la mayoría de los servicios es diurno, en días útiles y fines de semana y todos son direccionados por los equipos de Atención Primaria a la Salud. En relación a las demandas de cuidados, todos los niños necesitan de rehabilitación psicomotora y social, el 72,2% utilizan oxigenoterapia, el 66,6% de gastrostomía, el 55,5% de traqueotomía y el 50% de ventilación mecánica.

**Conclusión:**

El cuidado domiciliar presenta complexidades provenientes de condiciones crónicas y uso de dispositivos tecnológicos. Así, equipos de atención domiciliar son fundamentales en la asistencia y capacitación de familiares/cuidadores para el cuidado domiciliar a los niños.

## INTRODUCTION

In Brazil, children with special health care needs (CSHCN) are understood as the group of children who have one or more chronic health conditions, demanding continuous, temporary and, sometimes, permanent care, requiring a greater number of health visits than a healthy child. The CSHCNs require care related to neuropsychomotor development, and most are dependent on technological devices, on the continuous use of drugs, and have adaptation requirements in routine activities. The care of these children by the family requires support from health professionals to perform clinically complex procedures, including life support technologies management^(1,2)^.

A study carried out in the United States, between 2017 and 2018, revealed that about 18.5% of children and adolescents, aged from zero to 17 years old, had Special Health Care Needs (SHCN)^(3)^. In Brazil, there are still no official epidemiological data on CSHCN; however, a study carried out in a pediatric inpatient unit of a teaching hospital in southern Brazil showed that 44% of hospitalized children were CSHCN^(4)^.

Children who have a chronic health condition or who use some technological device need hospitalization for long periods, until the possibility of dehospitalization, in a safe and responsible way, based on a thorough and systematic planning, involving the hospital, the team that will take care of the child at home, and the family. Care, which was previously performed in the hospital, needs to be maintained in the home environment, on an ongoing basis, to ensure the child’s well-being and to avoid clinical condition worsening and consequent need for readmissions^(5)^. Given this, and considering the complexity and intensity of care demanded by CSHCN, due to the use of technological equipment and more complex procedures, the Home Care Service (*SAD*) becomes essential for assisting this group of children^(6)^.

Regarding Home Care (*AD*) in Brazil, the first reports appeared in 1960 and were strengthened from 1990 onwards. In 1998, Ordinance No. 2.416 was published, which established requirements for the accreditation of hospitals and criteria for performing home care. Between 2000 and 2006, specific ordinances were published for the Therapeutic Home Care program for Aids (*ADT-Aids*) and the program aimed at caring for the elderly. During this period, there were several experiences and projects in *AD* developed by municipal health departments or hospitals, however with no incentive policies or regulations for their operation^(7)^.

In 2006, continuing the regulation of *AD* in Brazil, ordinances and resolutions were published; however, few changes were perceived in health care. It was only in 2011 that the subject of *AD* was taken up again by the Ministry of Health (MS) and, in August 2011, Ordinance No. 2029, instituting *AD* within the Brazilian Public Health System (*SUS*), establishing rules for *SAD* registration and enablement, was published. In October of the same year, Ordinance No. 2.029 was revoked by Ordinance No. 2.527, which redefined *AD* within the scope of *SUS*. On November 8, 2011, the MS launched the Program *Melhor em Casa*, to expand home care developed by *SUS* ^(7)^.

Currently, *AD* is defined by Ordinance No. 825, of April 25, 2016, as a health care modality responsible for prevention, treatment, rehabilitation, palliation, and health promotion actions, provided at home, with a guarantee of continuity of care, and integrated into the Health Care Network (*RAS*). Thus, the SAD becomes a complementary service to the care provided in Primary Health Care (PHC) and in emergency services, substituting or complementing hospital admission, with care provided by Multiprofessional Home Care Teams (*EMAD*) and Support Multiprofessional Teams (*EMAP*)^(6)^.

Also, Ordinance No. 825/2016 divides *AD* into three modalities: AD1, AD2 and AD3. The AD1 modality includes users who require less frequent care, with assistance provided by PHC teams. Users who fit into the AD2 and AD3 modalities are those eligible for *SAD* because they have acute, acute-on-chronic, chronic-degenerative diseases, and need for weekly palliative care^(6)^.

Thus, the objectives of this study were: to characterize Home Care Services in the state of Santa Catarina, Brazil, and to know care demands of children with special health needs.

## METHOD

### Design of Study

Descriptive, exploratory study, with a quantitative approach, linked to the multicenter research “Production of care and validation of flow protocol for home care services for children with special health needs” that is being carried out in the states of Rio Grande do Sul (RS), Santa Catarina (SC), Paraná (PR), São Paulo (SP), Mato Grosso do Sul (MS), Paraíba (PB), and Maranhão (MA). In this study, data from the state of Santa Catarina will be discussed.

### Population

Eleven municipalities in the state of Santa Catarina were invited to participate in the study, that is, all those in which *SAD* was implemented and in full operation, enabled to operate an MS ordinance, as public services that are part of the program *Melhor em Casa*. The 11 municipalities are located in six of the eight regions that make up the state of SC, three in the region of Vale do Itajaí, two in the South region, two in the North region, two in the West region, one in the Greater Florianópolis region, and one in the Mountains region. The first contact with the *SAD* took place via telephone, aiming at explaining the study and making the invitation. The following criteria for selection of participants were adopted: being a health professional, working in *SAD* at the time of data collection, and the service providing home care for children. Thus, seven services participated in the study, as two justified not providing care to children and two were excluded from the research due to lack of response, after three attempts to contact them.

### Data Collection

It was held from April to July 2020, in which each *SAD* received a *link* via *Google forms,* containing the data collection instrument prepared by the research team, with questions about the following aspects: identification and organization of the service, identification of the participating professional, number of children served, age of children served, diagnoses and special health care needs of children, dynamics of home visits, and activities performed.

### Data Analysis and Treatment

The collected data were exported to the program *Excel* and organized into relative and absolute frequencies, building a matrix for the analysis of descriptive data.

### Ethical Aspects

The study followed the Resolution of the National Health Council no. 466/12, being approved by the Human Research Ethics Committee of the Universidade Estadual do Oeste do Paraná, protocol no. 3.477.776 of 07/31/2019 and its complementation report no. 3.928.387 of 03/23/2020. Following acceptance to participate in the research, and before starting data collection, the Free and Informed Consent Form (ICF) was obtained via *Google Forms.*

## RESULTS

In Table 1, data from characterization of *SAD*s in the State of Santa Catarina are presented.

**Table 1.** Characterization of Home Care Services in the state of Santa Catarina – Chapecó, SC, Brazil, 2020.

Characteristic		N	%
Professional interviewed	Nurse Another professional	4 3	57.1 42.9
Professional coordinating *SAD*	Nurse Another professional	6 1	85.7 14.3
Team	*EMAD* only *EMAD* and *EMAP*	3 4	42.9 57.1
Opening hours	Daytime Day and Night	5 2	71.4 28.6
Service on weekends	Yes No	5 2	71.4 28.6
Location where services are held	Residence Headquarters and residence	5 2	71.4 28.6
Services referring to *SAD*	PHC	7	100
	Public hospitals	6	85.7
	First Aid Service Units *(UPAs)* and oncology hospitals	3	42.9
Use the unique therapeutic design	Yes No	5 2	71.4 28.6
Has service protocols	Yes No	5 2	71.4 28.6
Methods used to train/guide CSHCN parents/caregivers at home	Verbal guidance Written guidance Demonstration	7 6 5	100% 85.7% 71.4%

As for home visits (HV) by the teams workers, all services reported that 100% of the professionals perform HV to children followed by *SAD*. Regarding the frequency with which the children are visited, six (85.7%) reported it was weekly and one (14.3%) visited more than once a week. Among the activities performed by *SAD*s’ workers, during the HVs, all services reported complying with the clinical evaluation and prescriptions. It should be noted that in six (85.7%) *SAD*s, professionals perform procedures that are later delegated to the caregiver, so that he/she can guarantee the continuity of care for the child at home.

Regarding the care of CSHCN by the *SAD*s, from May to July 2020, 18 children were attended by the *AD* teams, with 15 (83.3%) being under the age of six and three (16.6%) from six to 12 years. Among the main diagnoses, neurological or neuromuscular alterations stand out in 11 (61.1%) children, prematurity in three (16.6%), congenital malformation in three (16.6%), and cardiovascular conditions in one (5.5%).

In Table 2, the care demands of children with special health needs attended by the Home Care Services in the state of Santa Catarina are presented.

**Table 2.** Care demands of children with special health needs attended by Home Care Services in the state of Santa Catarina – Chapecó, SC, Brazil, 2020.

Care demands		N	%
Classification in AD	AD2 AD3	10 8	55.5 44.4
Need for psychomotor and social rehabilitation	Yes No	18 0	100 0.0
Use of gastrostomy	Yes No	12 6	66.6 33.3
Use of nasoenteral tube	Yes No	7 11	38.8 61.1
Use of tracheostomy	Yes No	10 8	55.5 44.4
Invasive and non-invasive mechanical ventilation	Yes No	9 9	50.0 50.0
Oxygen therapy	Yes No	13 5	72.2 27.7
Use of implanted/semi-implanted catheter and use of ostomies	Yes No	1 17	5.5 94.4
Use of food supplements	Yes No	14 4	77.7 22.2
Use of Drugs and differentiated care – for feeding, grooming, and dressing	Yes No	13 5	72.2 27.7

## DISCUSSION

In *AD*, actions are carried out, following the principles of equity of access, embracement, humanization, and integrality of care, developed by multiprofessional and interdisciplinary teams through clinical care practices, according to the user’s need, encouraging the participation of professionals, users, family, and caregivers in the actions and interventions carried out at home^(6)^. The nurse, a component of the multiprofessional team and working fully in the SAD, plays an important role in the construction of home care, performing activities directly related to patient care, also assuming the role of coordinating the AD team. In the present study, most SADs in SC are coordinated by nurses, a competence regulated by Resolution No. 464/2014 of the Federal Council of Nursing (COFEN)^(8,9,10)^.

A study carried out in Paraná also identified the nurse as the professional responsible for coordinating most services, thus developing a fundamental role in *AD*, which involves not only the function of coordinating the team and the care plan, but also giving, most of the time, the guidelines to users, caregivers, and family members^(11)^.

According to the ordinance regulating *SAD*, this service shall be constituted by the *EMAD* – main team, formed by physicians, nurses, nursing assistants/technicians, physical therapists, or social workers. It shall also have a support team for *EMAP* consisting of at least three professionals who can be a social worker, physical therapist, speech therapist, nutritionist, dentist, psychologist, pharmacist, or occupational therapist.

As for its denomination, *EMAD* can be type 1 when it is implemented in municipalities with a population greater than or equal to 40 thousand inhabitants and type 2 when the population is from 20 thousand to 39,999 inhabitants, or through grouping, in the case of those with less than 20 thousand inhabitants. In addition to the number of inhabitants, for the implementation of a *SAD*, the municipality shall have a reference hospital in the municipality or region and coverage of the Mobile Emergency Care Service (*SAMU* 192)^(6)^.

Thus, considering the population of the municipalities under study, the number of *EMAD*s implanted is in accordance with the regulations. As for *EMAP*, three municipalities have not implemented it yet. It is believed that the implementation of the support team in these municipalities would contribute to the planning and execution of actions together with professionals from different areas^(6)^.

However, despite the requirements for the implementation of *SAD*s in the municipalities, we can observe that of the states participating in the multicentric research, as well as in SC, the number of *SAD*s implanted is still low, and the state of Rio Grande do Sul (RS) has 15 *SAD*s, Maranhão with 11, Paraíba with nine, Paraná with eight, and Mato Grosso do Sul with four. The exception is the state of São Paulo, which has 77 *SAD*s; however, the number of municipalities in this state is also higher when compared to the state of SC. This way, it is believed that conducting research and disseminating data on services already implemented will encourage other municipalities to know and implement *SAD*, given its importance in *RAS* for the care of patients at home level^(6)^.

Regarding *SAD*s opening hours, in the present study most follow the regulation, performing the consultations on weekdays and on weekends. However, two *SAD*s do not attend on weekends, thus failing to comply with the recommendation and leaving users and family members with no assistance from the *AD* teams on those days. A similar reality was found in eight *SAD*s in the state of Paraná, with a predominance of daytime care from Monday to Friday and weekends, with only one service not being available on weekends^(11)^.

As for home care, most *SAD*s in the present study comply with this recommendation and provide care at the user’s home. Thus, through HV, professionals are able to perceive the family in their social environment and approach the person in an integral and individualized way, considering their reality. When carrying out the HV, the teams will face different family relationships permeated by affection, kindness, love, and also by conflicts and negligence. Faced with these different realities, the team needs to carry out an individualized approach to each family, aiming to create strategies to reestablish bonds and harmony and, thus, strengthen home care^(12)^.

Regarding the frequency of HVs, Ordinance No. 825^(6)^ recommends that they be weekly, as frequent monitoring helps to reduce or avoid hospitalizations. As for the *SAD*s in the study, we can observe that they all follow the regulations and carry out weekly visits to the children, since all the children, being monitored by them, fit into the AD2, AD3 modalities and use at least one technological device.

Regarding referrals to the *SAD*, it is recommended that it establishes flows with other *RAS* services, especially hospitals, emergency services, and PHC. The direct demand from users to *SAD* shall be avoided, since care needs to be shared among services. In the present study, *SAD*s reported receiving patients from PHC, hospitals, *UPAs,* and oncology hospitals, thus contributing to the dehospitalization and articulation of the service with *RAS* ^(6)^.

However, a study^(13)^ argues that the articulation of *AD* with RAS is a challenge, since many services are not aware of the role of *AD* in providing care to users at home. Thus, they identify the need for communication and training of health professionals in relation to the attributions of the *AD* teams and the functioning of *SAD*s.

This articulation difficulty was also identified in a study carried out in six *SAD*s in the state of RS, in which the participants identified only the hospital and the Primary Health Care Unit (*UBS*) as articulated services to the *SAD* and concluded that this may occur due to little knowledge, on the part of professionals, referring to this process of interaction^(14)^.

A study^(10)^ carried out with *SAD*s in the state of Minas Gerais revealed that the managers of these services recognized the need for dialogue with all points of the *RAS* as a guarantee of effective communication, to allow the continuity of care for users at home. Moreover, they understand that services need to talk about admission, discharge, hospital readmission flows due to worsening of the clinical condition and about the eligibility criteria, according to the recommendations defined in the current legislation^(6)^.

Communication among services can occur through referral and counter-referral. In that study, it was observed that there is effective communication between *SAD* and PHC, through the continuous accomplishment of these two processes. This exchange of information between health care points becomes fundamental to promote the integration of services, guaranteeing the continuity of care offered to users, thus facilitating access to all levels of care and improving the quality of care provided to the population^(15)^.

Consequently, if only one process is carried out, communication and comprehensive assistance to the user get compromised. A study carried out in three specialized comprehensive health care centers in a municipality in Paraíba, on the referral and counter-referral of children with chronic diseases, showed that, due to the complexity of the care required by children with chronic conditions, PHC workers refer them to the services specialized by filling out the specific form. However, after the consultation, the counter-referral to PHC was not performed; consequently, the mother understands that she no longer needs to return to the health unit of origin, leading to bond break between families and this point of the RAS^(16)^.

Another strategy used by *EMAD* and *EMAP* for care planning is the Singular Therapeutic Project (*PTS*), which consists of a set of therapeutic actions for a person or for the community. Its construction involves the participation of health professionals, users and family, being divided into four stages: diagnosis and case analysis; definition of actions and goals; tasks division; and revaluation^(17)^.

Regarding the use of the PTS, there is the experience report of a team that used the four stages, involving the participation of the Family Health Support Center (NASF), user, and family. They developed proposals for multidisciplinary interventions taking place concomitantly with the user’s follow-up period. At the end, they concluded that the discussion and elaboration of the PTS in a multiprofessional and interdisciplinary team is fundamental and contributes to the resolution of complex cases^(18)^.

In the present study, five of the seven *SAD*s use the *PTS*; however, data revealed that the services use general protocols for child care at home, as they do not have a specific protocol for the care of this public. In relation to this finding, studies^(19,20)^ point out the importance of adopting a specific protocol according to the population that will be assisted, aiming at planning care, facilitating professional decision-making, and benefiting the user. These studies reinforce the need for the *SAD*s that provide care to CSHCN to follow specific protocols for the care of this public, to fully meet the demands and needs.

Regarding the admission of CSHCN, it was evident that in most *SAD*s in SC it is carried out by workers from *EMAD*, with emphasis on the nurse who is usually responsible for the educational process with the families and for identifying the needs, strengths, and weaknesses in the execution of care. This finding corroborates a study^(21)^ which reaffirms the importance of nurses actively participating in the educational process of family members, to identify the child’s needs and the care that will be carried out at home, since CSHCN demand continuous and complex care. In addition, it is essential that health professionals welcome these families, understand the impact of caring for a CSHCN at home and, at the same time, enhance the skills of caregivers in relation to the care demanded by them, to stimulate the importance of the domain in the practice of procedures and techniques to be performed^(22)^.

Regarding the methods used by professionals to train parents and caregivers, a study carried out at the outpatient clinic of neuropediatric physiotherapy at a teaching hospital with families of CSHCN identified that the greatest difficulties reported by family members of children with neurological pathologies were: positioning, feeding, bathing, carrying, dressing, stimulating, and playing. In view of this situation, an illustrative guide was prepared to assist physical therapists in guiding family members and supporting verbal guidance^(23)^.

In the present study, *SAD* professionals also reported that they use verbal guidance associated with writing and demonstration to guide CSHCN family members, signaling the effectiveness of these strategies. One study^(2)^ presents simulation as a strategy adopted by nurses, still in a hospital setting, to train parents and caregivers of children who would be cared for at home. Training included explanation, observation, execution, supervision, collaboration, and performance evaluation, along with the observation about the need to favor the role of caregivers in the process of caring for the child, as well as their preparation to deal with possible complications at home. The researchers found this strategy to be effective in enabling caregivers and family members to care for a CSHCN.

Reinforcing the effectiveness of strategies involving simulation to train family members for care, a study developed in a simulation center of a public university in southern Brazil involved caregivers who evaluated training as positive and highlighted its importance to avoid errors that could worsen the child’s clinical condition. Upon completion, they expressed that they felt confident, assured, and more prepared to care for the child at home^(24)^.

In the present study, *SAD*s reported that all the children assisted by them need differentiated care, including administration of medication, food, technological devices, and psychomotor and social rehabilitation. A study carried out with 25 CSHCN in a hospital in the southern region of the country, on the classification of care demands, showed that 36% used some type of technology, 40% needed assistance in neuropsychomotor development, 92% were being followed up by some type of health care service, including physical therapist, speech therapist, nutritionist, and medical specialties, and 80% were using medication continuously^(4)^.

CSHCN followed by *SAD*s in SC have, as main diagnoses, neurological or neuromuscular alterations, prematurity, congenital and cardiovascular malformations, corroborating a study carried out in the state of PR, in which most CSHCN attended by *SAD*s had a diagnosis of cerebral palsy (25.7%), hydrocephalus (14.3%), prematurity (8.6%), and degenerative diseases (8.6%)^(11)^.

Studies from SC and PR corroborate the findings of a study carried out in Rio de Janeiro, which shows that Special Health Care Needs (SHCN) related to chronic diseases in children affect the respiratory, endocrine, cardiovascular, digestive, nervous, skeletal, and hematological immune systems. Furthermore, they lead to infections such as HIV/AIDS, consequently leading to changes in the way of caring for the child at home, generating demands related to modified usual care that, depending on the child’s needs, will imply adaptations of the home environment, creating a challenge for your caregivers^(25)^.

Research carried out in these three states revealed that the vast majority of children served by *SAD*s had a complex chronic condition and used technological devices. In view of this, it is worth noting that technological advances in the health area have caused important changes in child health care, especially leading to increased survival, allowing the transfer of care from the hospital environment to the home environment and preventing early deaths, which were common, due to serious illnesses such as prematurity, congenital malformations, and chronic conditions^(5)^.

Considering the increase in children with chronic health conditions and the use of technological devices to maintain life, the need to guide and train CSHCN parents/caregivers for home care is evident, in addition to the importance of having a multidisciplinary team to monitor families, especially in the first days after discharge, to help them adapt to the new reality.

The Nurse, being the professional that stands out in *EMAD*s, for being in frequent contact with the CSHCN family, needs to have a wide knowledge about the care demands of these children at home, to plan the discharge, prepare the family, and prepare the house to receive the child. Thus, carrying out studies that address this issue directly implies the quality of care provided and the guidelines that will be conveyed to the parents/caregivers by nursing professionals.

Further studies at the hospital level on the preparation of these families for hospital discharge, the limitations of health professionals in caring for CSHCN at home, and on communication among hospital teams and *SAD*s are suggested, aimed at a shared agreement on hospital discharge. Regarding the limitations of the study, the reduced number of *SAD*s implemented in the state of Santa Catarina stands out, in addition to two services not providing care for children, consequently generating a small sample.

## CONCLUSION

In the state of SC, the seven *SAD*s participating in the study, for the most part, are organized according to the recommendations of the current ordinance and work in a multidisciplinary team to meet the demands of the pediatric population. They play an important role in the child’s rehabilitation, adaptation to the use of technological devices, and in the guidance and training of parents and caregivers for the continuity of care with the child at home.

CSHCN followed by *SAD*s present complexities due to chronic conditions and the use of devices to maintain life, reinforcing that the training of caregivers/family members to take care of the child at home needs to be started as early as possible and remain continuous under supervision of the *EMAD* and *EMAP*, aiming at reducing children’s clinical complications and avoiding new hospitalizations.

It should also be noted that the study attributes to the nurse the coordination of most *AD* teams, also assuming an important role in the education and training of parents and caregivers for the care of CSHCN at home.

In addition, the study highlights important practices by the teams, which involve carrying out referral and counter-referral processes and the *PTS*, with the sharing of information through these actions bringing services, teams and users closer, strengthening and expanding child care in *RAS*.

## References

[1] Arrué AM, Neves ET, Magnago TSBS, Cabral IE, Gama SGN, Hökerberg YHM (2016). Translation and adaptation of the Children with Special Health Care Needs Screener to Brazilian Portuguese. Cad Saude Publica..

[2] Goes FGB, Cabral IE (2017). Discourses on discharge care for children with special healthcare needs. Rev Bras Enferm..

[3] HRSA Maternal e Child Health (2020). Children with Special Health Care Needs United States.

[4] Santos RP, Severo VRG, Kegler JJ, Jantsch LB, Cordeiro D, Neves ET (2020). Characterization of children with special health care Needsand caregivers in a teaching hospital. Ciência, Cuidado E Saúde..

[5] Carvalho MSN, Menezes AL, Cruz ADF, Maciel CMP (2019). Desospitalização de crianças com condições crônicas complexas: perspectivas e desafios.

[6] Brasil. Ministério da Saúde. Portaria nº 825, de 25 de abril de 2016 (2016). Redefine a Atenção Domiciliar no âmbito do Sistema Único de Saúde (SUS) e atualiza as equipes habilitadas. [Internet].

[7] Oliveira AV, Dias MB (2014). Home Care in Unified Health System (SUS): what Melhor em Casa Program represented?. Divulgação Em Saúde Para Debate..

[8] COFEN (2014). Resolução nº 0464 de 2014. Normatiza a Atuação da Equipe de Enfermagem na Atenção Domiciliar. Cofen [Internet].

[9] Andrade AM, Silva KL, Seixas CT, Braga PP (2017). Nursing practice in home care: an integrative literature review. Rev Bras Enferm..

[10] Castro EAB, Leone DRR, Santos CM, Neta FCCG, Gonçalves JRL, Contim D (2018). Home care organization with the Better at Home Program. Rev Gaúcha Enferm..

[11] Rossetto V, Toso BRGO, Rodrigues RM, Viera CS, Neves ET (2019). Development care for children with special health needs in home care at Paraná – Brazil. Escola Anna Nery..

[12] Sugiura S, Caceres N, Lacerda M, Tonin L, Rodrigues J, Nascimento J (2018). A vivência do contexto domiciliar por familiares e profissionais de saúde. Revista de Enfermagem da UFSM..

[13] Procópio LCR, Seixas CT, Avellar RS, Silva KL, Santos MLM (2019). Home Care within the Unified Health System: challenges and potentialities. Saúde em Debate..

[14] Weykamp JM, Siqueira HCH, Cecagno D, Medeiros AC, Paula SF, Pedroso VSM (2019). Home Care Service and Health Care Networks. Revista de Pesquisa Cuidado é Fundamental Online..

[15] Oliveira CCRB, Silva EAL, Souza MKB (2021). Referral and counter-referral for the integrality of care in the Health Care Network. Physis..

[16] Vaz EMC, Brito TS, Santos MCS, Lima PMVM, Pimenta EAG, Collet N (2020). Referral and counter-referral of children in chronic condition: perception of mothers and secondary care professionals. Revista Enfermagem UERJ.

[17] Baptista JA, Camatta MW, Filippon PG, Schneider JF (2020). Singular therapeutic project in mental health an integrative review. Rev Bras Enferm..

[18] Moura AMLS, Santos EVL (2019). Singular therapeutic project: multidisciplinary team in complex case management. J Prev Health Promo..

[19] Duarte TCR, Oliveira E M, Morais SLA (2019). Protocol for the evaluation and classification of Pediatric patients according to the nursing team demand level. Revista Enfermagem Atual In Derme..

[20] Araújo MCC, Acioli S, Neto M, Silva HCDA, Bohusch G, Rocha FN (2020). Nursing protocols in primary health care: instrument for quality of care. Cogitare Enfermagem..

[21] Precce ML, Moraes JRMM (2020). Educative process with relatives of children with special health needs in the hospital-home transition.. Texto & Contexto – Enfermagem..

[22] Torquato RC, Rovere GP, Pitombeira MGV, Pereira AS, Santos LKX (2020). Preparation for the care of children with chronic diseases: the perception of caregivers. Rev Rene..

[23] Souza JS, Knobel KAB (2019). Guia ilustrado de orientações a cuidadores de crianças com deficiências neuromotoras.. ConScientia e Saúde..

[24] Silva APM, Pina JC, Rocha PK, Anders JC, Souza AIJ, Okido ACC (2020). Training of caregivers of children with special healthcare needs: simulation contributions. Texto & Contexto – Enfermagem..

[25] Cabral IE, Motta IS, Pimentel TGP, Corrêa MPO, Arrué AM, Neves ET (2020). Demands of children with special health care needs in primary care in Rio de Janeiro. Ciência, Cuidado e Saúde..

